# Guide to the Examination of the Urine

**Published:** 1886-03

**Authors:** 


GUIDE TO THE EXAMINATION OF THE URINE. By Hoffman and Ultzman. Translated by F. Forshheimer, Professor of Physiology at the Medical College of Ohio. Second edition. Woodruff, Cox & Co., Cincinnati, 1886. 12 mo., pp., 251.

This is a thorough, concise and highly practical little book, and the translator deserves credit, not only for his excellent translation, but for the fact that he has rendered available to American physicians and students the work of such authorities as Ultzman and Hoffman. The chapter on "Diagnosis of Diseases of the Urinary Apparatus," in which are presented the chemical and microscopical characteristics of the urine under different pathological conditions, is most valuable. The illustrations are good and the elaborate index at the end of the book adds greatly to its value as a book of reference.

